# Broad learning for early diagnosis of Alzheimer's disease using FDG-PET of the brain

**DOI:** 10.3389/fnins.2023.1137567

**Published:** 2023-03-13

**Authors:** Junwei Duan, Yang Liu, Huanhua Wu, Jing Wang, Long Chen, C. L. Philip Chen

**Affiliations:** ^1^College of Information Science and Technology, Jinan University, Guangzhou, China; ^2^Guangdong Provincial Key Laboratory of Traditional Chinese Medicine Informatization, Jinan University, Guangzhou, China; ^3^Department of Nuclear Medicine and PET/CT-MRI Centre, The First Affiliated Hospital of Jinan University, Guangzhou, China; ^4^School of Computer Science, Guangdong Polytechnic Normal University, Guangzhou, China; ^5^Department of Computer and Information Science, Faculty of Science and Technology, University of Macau, Taipa, Macau SAR, China; ^6^School of Computer Science and Engineering, South China University of Technology, Guangzhou, China

**Keywords:** Alzheimer's disease, PET, broad learning system, neural network, computer-aided diagnosis

## Abstract

Alzheimer's disease (AD) is a progressive neurodegenerative disease, and the development of AD is irreversible. However, preventive measures in the presymptomatic stage of AD can effectively slow down deterioration. Fluorodeoxyglucose positron emission tomography (FDG-PET) can detect the metabolism of glucose in patients' brains, which can help to identify changes related to AD before brain damage occurs. Machine learning is useful for early diagnosis of patients with AD using FDG-PET, but it requires a sufficiently large dataset, and it is easy for overfitting to occur in small datasets. Previous studies using machine learning for early diagnosis with FDG-PET have either involved the extraction of elaborately handcrafted features or validation on a small dataset, and few studies have explored the refined classification of early mild cognitive impairment (EMCI) and late mild cognitive impairment (LMCI). This article presents a broad network-based model for early diagnosis of AD (BLADNet) through PET imaging of the brain; this method employs a novel broad neural network to enhance the features of FDG-PET extracted *via* 2D CNN. BLADNet can search for information over a broad space through the addition of new BLS blocks without retraining of the whole network, thus improving the accuracy of AD classification. Experiments conducted on a dataset containing 2,298 FDG-PET images of 1,045 subjects from the ADNI database demonstrate that our methods are superior to those used in previous studies on early diagnosis of AD with FDG-PET. In particular, our methods achieved state-of-the-art results in EMCI and LMCI classification with FDG-PET.

## 1. Introduction

Alzheimer's disease (AD) cannot be diagnosed until obvious symptoms appear in the patient, but studies have found that patients with AD show abnormalities in regional metabolism before brain structure changes occur (Jagust et al., 2006). Fluorine 18 (18F) fluorodeoxyglucose (FDG) positron emission tomography (PET) is a non-invasive nuclear medicine imaging technique that can indicate the metabolic activity of tissues and organs (Marcus et al., 2014; Bouter et al., 2019; Levin et al., 2021). FDG-PET may detect the onset of certain diseases earlier than other imaging tests (Brown et al., 2014). FDG-PET is regarded as an effective biomarker for earlier diagnosis of AD (Chételat et al., 2020). The onset of Alzheimer's disease is insidious and slow, and it can be divided into three stages: cognitively normal (CN), mild cognitive impairment (MCI), and Alzheimer's disease (AD). Patients with AD tend to show hypometabolism on 18F-FDG-PET scan in the regions of the posterior cingulate, parietotemporal cortices, and frontal lobes, while patients with MCI often show posterior cingulate and parietotemporal hypometabolism with variable frontal lobe involvement (Mosconi et al., 2008; Kobylecki et al., 2015). However, the difference between the two stages in FDG-PET is difficult to distinguish with the naked eye or through pattern recognition-based decisions made *via* qualitative readings. Because the disease involves a wide continuous spectrum, from normal cognition to MCI to AD, MCI can also be subdivided into early MCI (EMCI) and late MCI (LMCI) (Jessen et al., 2014).

Machine learning approaches can effectively extract features that are difficult to find with the naked eye and can outperform professional clinicians in certain imaging diagnosis problems (Zhang et al., 2020). A number of studies have already experimented with unsupervised learning (Suk and Shen, 2013), adversarial learning (Baydargil et al., 2021), and multi-scale learning (Lu et al., 2018) techniques in AD-related PET image analysis. These methods have achieved good results in classification of CN, MCI, and AD, but few studies have explored the refined classification of early EMCI and late LMCI.

Currently, deep learning-based approaches have been applied in early diagnosis of AD (Suk and Shen, 2013; Lu et al., 2018). Nevertheless, there are still many issues remaining in deep learning, such as gradient explosion and vanishing gradients, which limit the depth in terms of number of layers in the network or its fitting ability; some researchers have proposed residual learning (He et al., 2016) as a way to alleviate this problem. The broad learning system (BLS) is one kind of neural network without deep structure. BLS provides better fitting ability by increasing the number of network nodes horizontally and obtains solutions *via* pseudoinverse, with no need for an iterative backpropagation process. However, BLS obtains a feature representation of input data through random projection, which may result in too much redundant information that could influence the performance of the BLS model. Some researchers have experimented with variations of BLS that use other models as feature extractors in the feature mapping layer (Feng and Chen, 2018; Du et al., 2020; Jara-Maldonado et al., 2022; Wu and Duan, 2022). In this article, we propose a novel BLS-based method, in which we use grouped convolution layers to extract the features from slice groups in the first stage, and then these features are fed into a broad learning model for further feature enhancement.

This study proposes a machine learning model based on BLS to predict the clinical diagnosis in patients using 18F-FDG-PET of the brain. We attempted to predict patients' classifications as AD, MCI, or CN, and (within the category of MCI) as EMCI or LMCI. The hypothesis was that the broad learning-based model would be able to detect regional metabolic abnormalities caused by pathology, which are difficult to observe on clinical review, and improve the accuracy of individual diagnosis.

## 2. Materials and methods

### 2.1. Data acquisition

Data used in the preparation of this article were obtained from the Alzheimer's Disease Neuroimaging Initiative (ADNI) database (adni.loni.usc.edu). ADNI was launched in 2003 as a public–private partnership, led by Principal Investigator Michael W. Weiner, MD. The primary goal of ADNI has been to test whether serial magnetic resonance imaging (MRI), PET, other biological markers, and clinical and neuropsychological assessment can be combined to measure the progression of MCI and early AD.

In our study, we analyzed a total of 2,298 FDG-PET imaging studies of 1,045 patients obtained from ADNI. The datasets contained images of subjects of different ages. In ADNI 1, the subjects were grouped into three classes: CN, MCI, and AD. However, in ADNI 2/GO, the MCI stage was subdivided into EMCI and LMCI. To be classified as CN, subjects must have no memory complaints and be non-demented. To be classified as having MCI, subjects must have a Mini-Mental State Examination (MMSE) score between 24 and 30; the activities of daily living must be preserved, and dementia must be absent. Finally, to be classified as having AD, subjects must be clinically diagnosed as such, with an MMSE score between 20 and 26 (Jack Jr et al., 2008). Demographic information on our dataset is presented in Table 1. A total of 80% of the data (1,851 imaging studies, 598 patients) were used for model training. The remaining 20% (447 imaging studies; no repeat studies of the same subjects in the test set) were used for model testing, from which an additional test set (74 imaging studies for AD vs. MCI vs. CN classification and 45 imaging studies for EMCI vs. LMCI classification) was selected for validation by professional radiologists.

**Table 1:** Demographics of datasets.

			**Average age** ^*^	
**Clinical diagnosis**	**No. of Patients**	**No. of imaging studies**	**Men**	**Women**
AD	297	541	76.47 ± 7.57 (56–92)	75.11 ± 7.63 (55–92)
MCI	196	616	77.79 ± 7.01 (57–92)	74.82 ± 7.95 (57–96)
CN	242	627	77.12 ± 5.41 (62–91)	76.93 ± 6.37 (60–96)
Total	735	1,784	77.18 ± 6.75 (56–92)	75.76 ± 7.31 (55–96)
EMCI	152	265	73.89 ± 6.85 (56–90)	72.40 ± 8.40 (55–92)
LMCI	158	249	74.70 ± 7.37 (56–94)	71.80 ± 7.80 (55–91)
Total	310	514	74.27 ± 7.10 (56–94)	72.09 ± 8.11 (55–91)

AD, Alzheimer's disease; MCI, mild cognitive impairment; CN, cognitively normal.

* Data in parentheses are the range.

### 2.2. Data processing

**Figure 1:**
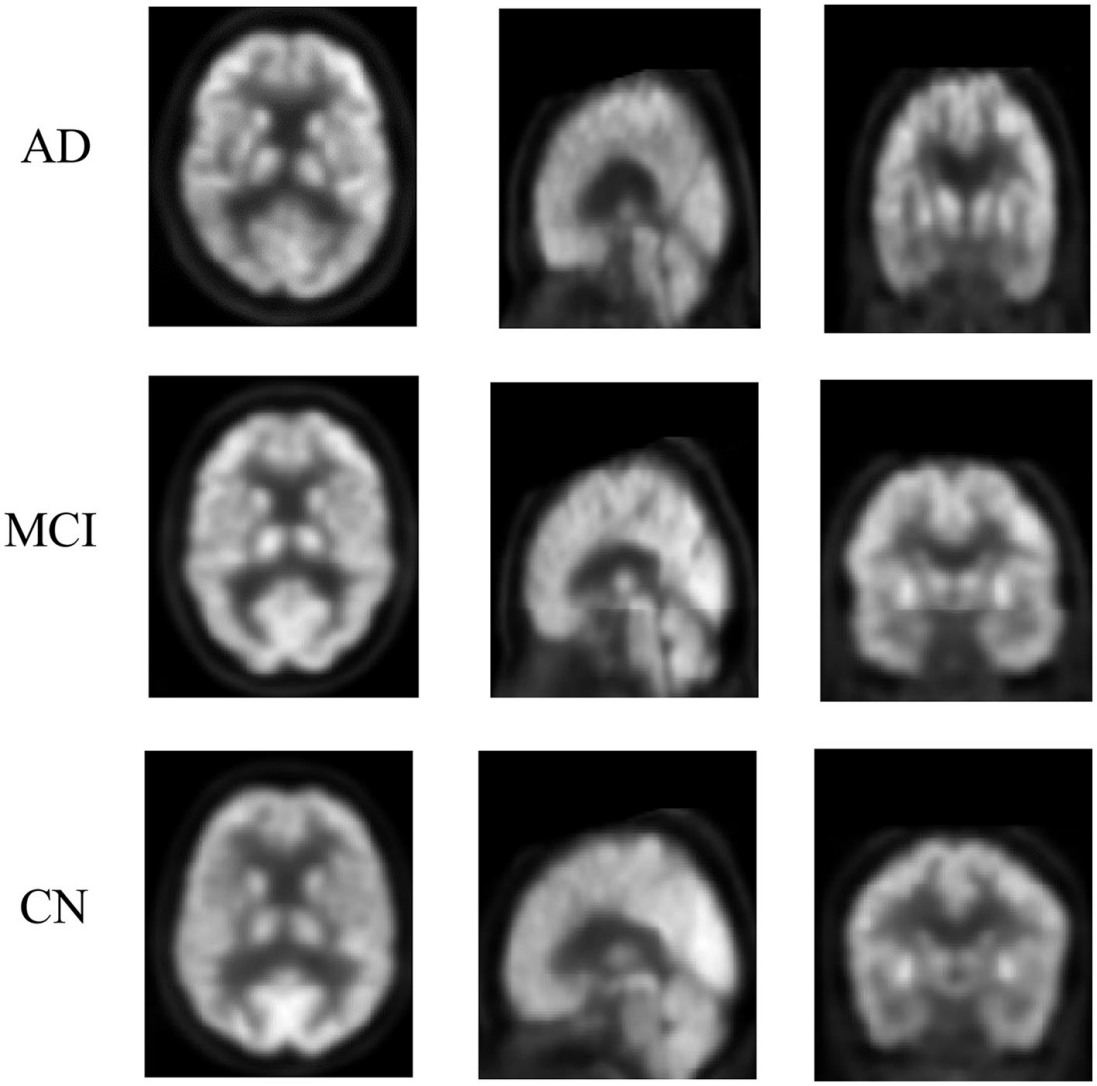
Example of FDG-PET imaging from ADNI. Each row represents a PET imaging slice on a three-plane view. The three rows are: a 73-year-old man with AD, an 81-year-old woman with MCI, and a 71-year-old man without MCI/AD. The difference between them is difficult to identify with the naked eye.

For the purpose of eliminating differences between images acquired from various systems, FDG-PET images in ADNI have undergone a series of preprocessing steps, intensity normalization, and conversion to a uniform isotropic resolution of 8 mm full width at half maximum. We selected the processed images from ADNI; our method does not require any specific pre-defined ROI or VOI as traditional machine learning methods do. All 3D images were resampled to a size of 160 × 160 × 96; we treated the images as a series of 2D slices and removed slices with all-zero intensity on both sides, then divided the image into four groups of slices at equal intervals, with each group containing 23 slices. All processing steps were conducted in Python (version 3.8) using the packages scipy (http://www.scipy.org) and numpy (https://numpy.org/). Figure 1 shows a single slice, viewed on three planes.

### 2.3. Model development

Despite the good learning ability of deep neural networks, they are easy to overfit on small datasets and their training is also time-consuming. The BLS is a lightweight network with a broad structure proposed by Chen and Liu (2017). The inspiration for its design comes from a random vector functional link neural network (RVFLLNN) (Pao and Takefuji, 1992; Chu et al., 2019; Gong et al., 2021). It can obtain a globally optimal solution using a ridge regression algorithm during training. without an iterative backpropagation process, meaning that its training is fast and efficient. The detailed description of the BLS is illustrated in the Supplementary material. Based on the BLS, we propose a broad network-based model for early diagnosis of AD (BLADNet) through PET imaging of the brain.

**Figure 2:**
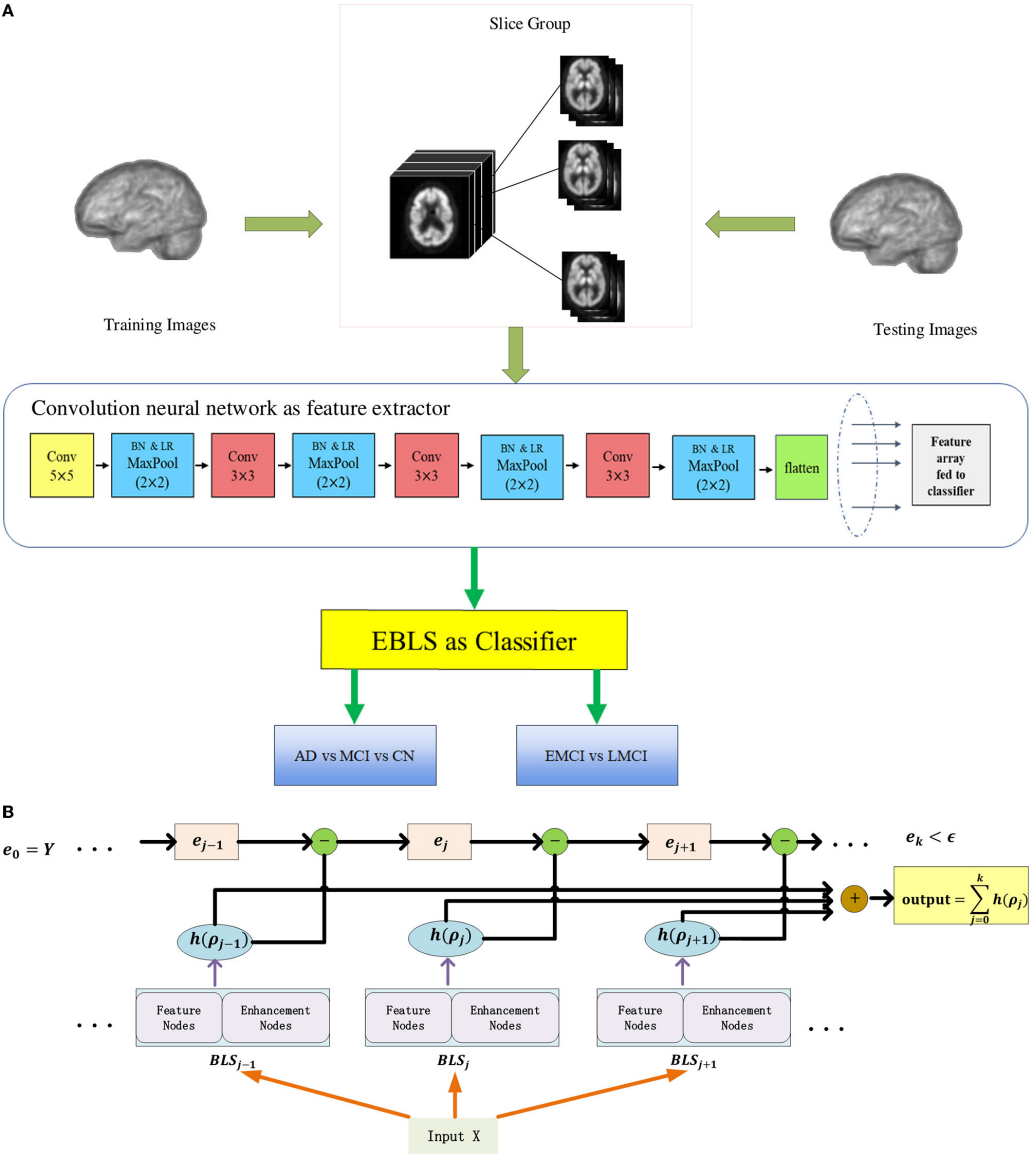
Each 3D image is decomposed into groups of 2D slices at equal intervals. In the first stage, deep convolutional features are extracted from each group by 2D CNN. In the second stage, all features from each slice group are concatenated to form a compact feature vector, and fed to EBLS for final prediction. **(A)** The overall architecture of BLADNet. **(B)** The detailed structure of EBLS.

Figure 2 illustrates the overall architecture of BLADNet. The framework consists of two stages. In the first stage, we use a 2D CNN for automated feature learning from each group of slices rather than directly using a 3D CNN, which reduces the number of parameters to be learned. In the second stage, the features extracted from each group in the previous step are concatenated to form a compact sequence feature; then, the Extreme Broad Learning System (EBLS), based on a broad neural network, is used to enhance the features from 2D CNN and carry out the final classification. A detailed description of the EBLS is provided in the Supplementary material^1^. Our model was developed in Python using the packages numpy and pytorch (https://pytorch.org/, version 1.7.1). All experiments were conducted using a computer with a Linux operating system (Ubuntu 18.04). The computer was equipped with a CPU (Intel(R) Core (TM) i9-9980XE, 3.00 GHz), 64 GB of DDR4 SDRAM, and GPU (GeForce RTX 3080) with CUDA Version 11.2 and cuDNN Version 9.1.85.

### 2.4. Model evaluation and statistical analysis

We performed the experiments of AD vs. MCI vs. CN classification as in previous studies on data from ADNI 1, and also performed additional experiments of refined classification between EMCI and LMCI on data from ADNI 2/GO. All data were randomly shuffled before being spli into the training set and test set. In all experiments, we used 80% of the samples for training and 20% of the samples for testing. In the experiment, we regard each category as positive samples respectively, the rest as negative samples, and then calculate metrics. We used accuracy, sensitivity, and specificity as metrics to evaluate classification performance. All metrics were calculated under a default threshold value of 0.5. We also plotted the ROC curve of all experiments and calculated the corresponding AUC.

**Figure 3:**
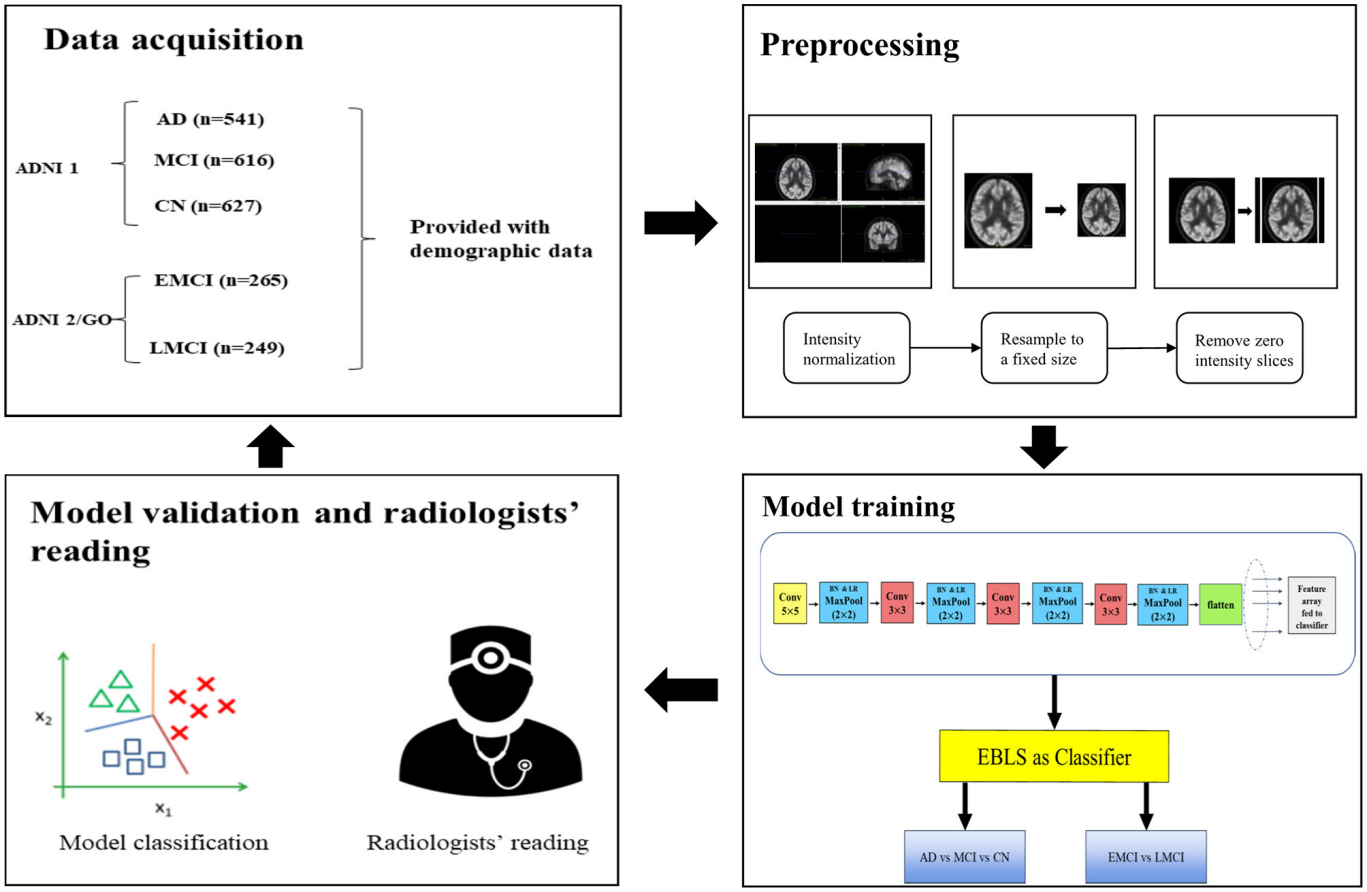
Main steps of the experiment. The data set was split into training and test sets at a ratio of 8:2. In the validation step, the radiologists were provided with demographic information to aid their readings.

Two board-certified professional radiologists working in a department of brain imaging and nuclear medicine (radiologist 1: HLZ, with 8 years of experience in brain imaging reading for AD diagnosis; radiologist 2: HHW, with 6 years of experience in brain imaging reading for AD diagnosis) were asked to give their diagnostic impressions of a dataset that was not used for model training. For each case, the radiologists were provided with the patient's age, gender, and MMSE score as additional information for validation. To validate the performance of the proposed model and the professional readings of radiologists, we compared the performance of our proposed model with that of the radiologists' interpretations. The main steps of the experiment are shown in Figure 3.

## 3. Experimental results

### 3.1. Results of model training

**Figure 4:**
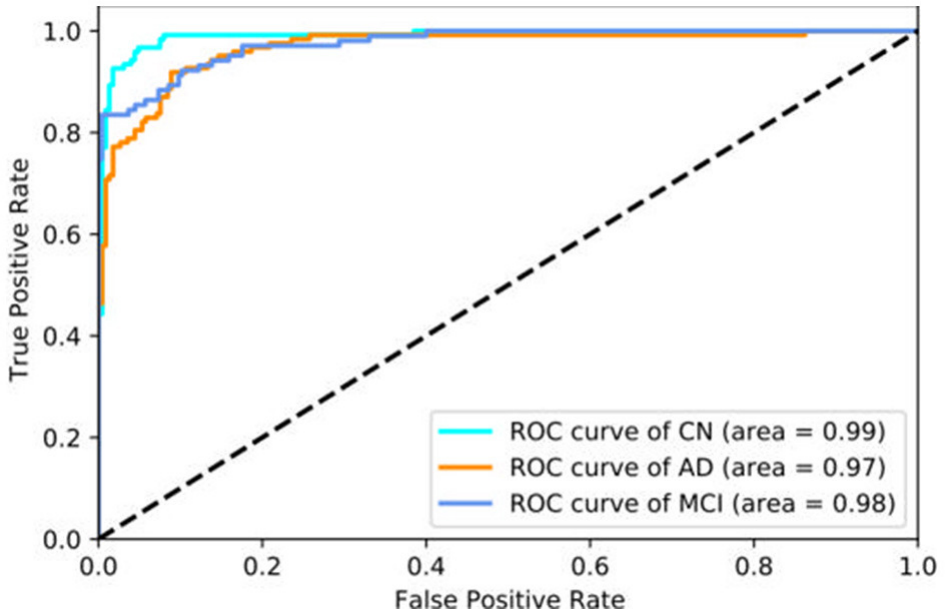
ROC curve of our method tested on the ADNI data set. The ROC curve labeled AD represents the model's performance in distinguishing AD vs. all other classes, the other curves represent the equivalent objective. The AUC is larger for CN than for the other classes, which indicates that our model can distinguish healthy subjects from patients with AD/MCI more successfully than other classifications.

The prediction results of the broad network-based model are shown in Table 2. For classification of AD, MCI, and CN samples, sensitivity was 92.16 (94 of 102), 89.34 (109 of 122), and 95.16% (118 of 124), respectively; specificity was 97.56 (240 of 246), 95.58 (216 of 226), and 95.09% (213 of 224), respectively; and precision was 94.00 (94 of 100), 91.60 (109 of 119), and 91.47% (118 of 129), respectively. The ROC curves of our model, trained on 80% of the ADNI data and tested on the remaining 20%, are shown in Figure 4. The AUC in prediction of AD, MCI, and CN was 0.97, 0.98, and 0.99, respectively. The AUC for CN was the highest, indicating that our model can distinguish healthy subjects from patients with AD/MCI.

**Table 2:** Comparison of performance between our model and radiology readers in prediction of AD, MCI, and CN.

**Our method on ADNI test set**	Sensitivity (%)^*^	Specificity (%)^*^	Precision (%)^*^	**F1 score (%)**	**No. of imaging studies**
AD	92.16 (94/102)	97.56 (240/246)	94.00 (94/100)	93.06	102
MCI	89.34 (109/122)	95.58 (216/226)	91.60 (109/119)	90.46	122
CN	95.16 (118/124)	95.09 (213/224)	91.47 (118/129)	93.28	124
**Radiologist 1**					
AD	51.85 (14/27)	57.45 (27/47)	41.76 (14/34)	45.9	27
MCI	29.41 (5/17)	80.70 (46/57)	31.25 (5/16)	30.3	17
CN	46.67 (14/30)	77.27 (34/44)	58.33 (14/24)	51.85	30
**Radiologist 2**					
AD	37.04 (10/27)	72.34 (34/47)	43.48 (10/23)	40	27
MCI	35.29 (6/17)	63.16 (36/57)	22.22 (6/27)	27.27	17
CN	46.67 (14/30)	77.27 (34/44)	58.33 (14/24)	51.85	30

AD, Alzheimer's disease; MCI, mild cognitive impairment; CN, cognitively normal.

* Data in parentheses are raw data used to calculate the percentage.

The results for EMCI and LMCI prediction are shown in Table 3. In this experiment, we treated LMCI as the positive class and EMCI as the negative class. Sensitivity was 81.63% (40 of 49) and specificity was 85.19% (46 of 54). Similar to the AD vs. MCI vs. CN experiment, the specificity of the model was much higher than the sensitivity, indicating that our model was better than radiologists at identifying healthy subjects.

**Table 3:** Comparison of performance between our model and radiology readers in prediction of EMCI and LMCI.

	Sensitivity (%)^*^	Specificity (%)^*^	Precision (%)^*^	**F1 score (%)**	**No. of imaging studies**
Our method on test set 2	81.63 (40/49)	85.19 (46/54)	83.33 (40/48)	82.47	103
Radiologist 1	84.00 (21/25)	25.00 (5/20)	58.33 (21/36)	68.85	45
Radiologist 2	76.00 (19/25)	30.00 (6/20)	58.33 (19/33)	65.52	45

EMCI, early mild cognitive impairment; LMCI, late mild cognitive impairment.

* Data in parentheses are raw data used to calculate the percentage.

### 3.2. Model interpretation: t-SNE plot

**Figure 5:**
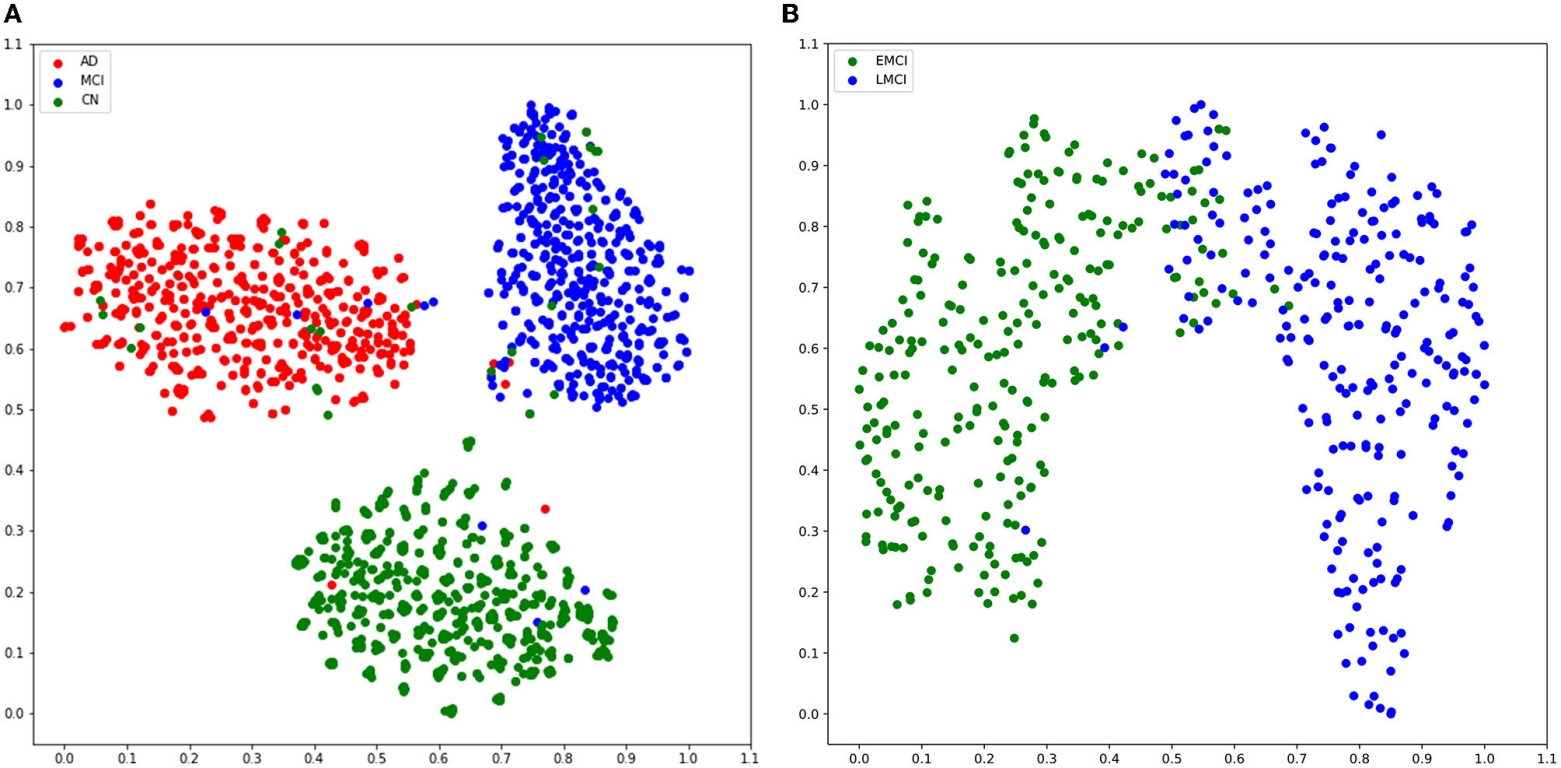
Scatter plot of all data after dimension reduction by t-SNE. **(A)** Visualization of dimension reduction for AD, MCI, and CN. **(B)** Visualization of dimension reduction for EMCI and LMCI.

We used the t-SNE algorithm to reduce the dimensionality of the features extracted from the convolutional network and projected them into a two-dimensional space for visualization. As shown in Figure 5A, for the AD vs. MCI vs. CN experiment, there were obvious boundaries between the three categories. Moreover, only a few samples from other categories were scattered within the CN category, indicating that the model has a better screening ability for healthy cases than for patients. Similarly, as shown in Figure 5B, for EMCI and LMCI classification, the model divided the samples very successfully into two clusters. Although a few cases were mixed in the junction of the two clusters, which indicates that there is a transition stage from EMCI to LMCI, our model could distinguish the two stages well.

### 3.3. Comparison of model predictions with state-of-the-art methods

Recently, a substantial amount of work has been carried out exploring the application of machine learning approaches to AD prediction using brain imaging. Most of these studies have used structural imaging of the brain, with few studies using functional imaging, specifically 18F-FDG-PET. Some researchers have attempted to analyze 18F-FDG-PET for AD predictions, but these studies have yielded limited success (Liu et al., 2018; Lu et al., 2018; Pan et al., 2018; Ding et al., 2019; Huang et al., 2019; Hamdi et al., 2022). Tables 4, 5 summarize state-of-the-art deep learning methods for prediction of AD using 18F-FDG-PET imaging. Most of the methods investigated can only discriminate AD from CN or MCI from CN, while our method can classify patients at different stages of AD with higher sensitivity and specificity. In addition, compared with these methods, we used a larger test set in our experiments, which demonstrates the superior generalization ability of our method.

**Table 4:** Summary of state-of-the-art methods for prediction of Alzheimer's disease (AD) using 18F-FDG-PET imaging.

**References**	**Summary of method**	**Dataset specifications**	**Sensitivity**	**Specificity**	AUC^*^
Ding et al. (2019)	Inception V3 network pre-trained on ImageNet	484 AD, 861 MCI, 764 non-AD/MCI scans from ADNI	AD 81%	94%	0.92
			MCI 54%	68%	0.63
			Non-AD/MCI 59%	75%	0.73
Huang et al. (2019)	3D VGG network	647 AD, 731 CN, 767 MCI 18F-FDG-PET scans from ADNI	AD vs. CN 90.24%	87.77%	0.9269
Lu et al. (2018)	A multiscale deep neural network	226 AD and 304 18F-FDG-PET scans from ADNI	AD vs. CN 91.54%	95.06%	NA
Liu et al. (2018)	A combination of 2D CNN and RNN	93 AD, 146 MCI, 100 CN scans from ADNI	AD vs. CN 91.4%	91%	0.953
			MCI vs. CN 78.1%	80%	0.839
Hamdi et al. (2022)	A 2D CNN network	220 AD, 635 CN FDG-PET scans from ADNI	AD vs. CN 94%	96%	0.95
Pan et al. (2018)	SVM	94 AD, 88 MCI, 90 CN subjects from ADNI	AD vs. CN 92.78%	91.38%	0.9598
			MCI vs. CN 84.20%	82.83%	0.8893
Current study	A broad learning-based network	541 AD, 616 MCI, 627 FDG-PET imaging studies from ADNI	AD 92.16%	97.56%	0.97
			MCI 89.34%	95.58%	0.98
			Non-AD/MCI 95.16%	95.09%	0.99

We report sensitivity, specificity, and area under the curve (AUC) for all these methods.

AD, Alzheimer's disease; MCI, mild cognitive impairment; CN, cognitively normal.

* A value of NA indicates that this result is not reported in the literature.

**Table 5:** Comparison of performance between our model and other existing methods in prediction of EMCI and LMCI.

**References**	Sensitivity (%)^*^	Specificity (%)^*^	F1 score (%)^*^	**Dataset specifications**
Singh et al. (2017)	64.82%	NA	0.6844	178 EMCI, 158 LMCI
Nozadi et al. (2018)	72.50%	79.20%	NA	164 EMCI, 189 LMCI
Forouzannezhad et al. (2020)	61.50%	64.3%	NA	296 EMCI, 193 LMCI
Ours	81.63%	85.19%	82.47%	265 EMCI, 249 LMCI

EMCI, early mild cognitive impairment, LMCI, late mild cognitive impairment.

* A value of NA indicates that this result is not reported in the literature.

### 3.4. Comparison of model predictions with professional radiologists

As shown in Table 2, two radiologists gave their interpretations of a test set. For radiologist 1, the sensitivity results for MCI, AD, and CN were 51.85 (14 of 27), 29.41 (5 of 17), and 46.67% (14 of 30), respectively; the specificity results were 57.45 (27 of 47), 80.70 (46 of 57), and 77.27% (34 of 44), respectively; and the precision results were 41.76 (14 of 34), 34.25 (5 of 16), and 58.33% (14 of 24), respectively. For radiologist 2, sensitivity for MCI, AD, and CN was 37.04 (10 of 27), 35.29 (6 of 17), and 46.67% (14 of 30), respectively; specificity was 72.34 (34 of 47), 63.16 (36 of 57), and 77.27% (34 of 44), respectively; and precision was 43.48 (10 of 23), 22.22 (6 of 27), and 58.33% (14 of 24), respectively. It can be observed that the prediction results of our proposed model were better than those of the radiologist, which indicates that the model was able to find lesions that were difficult to observe with the naked eye. It is also worth noting that although the two radiologists obtained the same results in their evaluations of healthy cases, patients with MCI and AD were difficult to evaluate.

Table 3 reports reader performance on prediction of EMCI vs. LMCI. For radiologist 1, the results in terms of sensitivity, specificity, and precision were 84.00 (21 of 25), 25.00 (5 of 20), and 58.33% (21 of 36), respectively. For radiologist 2, the results were 76.00 (19 of 25), 30.00 (6 of 20), and 58.33% (19 of 33), respectively. Although radiologists had higher sensitivity in this scenario, their specificity was very low; this is because radiologists tend to predict cases as LMCI. In contrast, our model was able to achieve high specificity under high sensitivity.

## 4. Discussion

With the aging of the population, the number of patients with AD is continuously increasing. However, research on a cure for AD has been slow, and the focus of research has shifted to the early diagnosis of AD, so that early prevention measures can delay the progression of the disease. However, early identification of patients at the prodromal stage of AD is still a challenging problem. The broad neural network-based model can identify patients with AD at different stages with high sensitivity and specificity. In addition, in identifying patients at the EMCI or LMCI stage, the proposed model is able to achieve high sensitivity under high specificity; notably, it outperformed professional radiologist readers, achieving higher sensitivity and specificity.

Previous research has studied the specific pattern of hypometabolism that can be observed in FDG-PET of patients with AD. Bilateral temporo-parietal hypometabolism has been found to be a dominant pattern related to clinically confirmed AD (Hoffman et al., 2000). Other studies have demonstrated that, as the disease progresses, FDG uptake is reduced, especially in the frontal, parietal, and lateral temporal lobes (Ossenkoppele et al., 2012). However, FDG-PET is not a definitive imaging biomarker for AD and MCI. Substantial previous efforts have been devoted to attempts to develop computer-aided methods of diagnosis of AD *via* other modalities, but few studies have been conducted involving attempts to applying machine learning approaches to classify patients with AD by FDG-PET alone. Previous attempts to identify MCI have resulted in limited sensitivity (81% for AD, 54% for MCI) and specificity (Ding et al., 2019). In addition to prediction of AD, our model performs refined classification of EMCI vs. LMCI, achieving sensitivity of 81.63% and specificity of 85.19% in doing so. Compared to previous studies, the key advantages of our model are as follows. First, due to the incremental learning ability of BLS, our model can be dynamically updated without retraining from scratch if new imaging studies are added; our EBLS model can further extend the incremental learning ability of BLS by adding new BLS blocks dynamically. In addition, our model exhibits better performance in the identification of the early stage of AD, which is of great significance for the diagnosis of AD, because early identification of AD facilitates early intervention in the progression of the disease. There are also some limitations to our model in that the training needs to be completed in two stages, and the process is complicated. In addition, training a convolution layer from scratch for the first time is still time-consuming work, and the BLS model in the second stage depends on the quality of feature extraction in the convolution layer.

Because of deep structure, deep learning models are very good at capturing abstract and intrinsic features of images. However, the problems existing in deep learning models, such as gradient explosion and vanishing gradients, usually limit the possibility of deepening the networks of deep learning models indefinitely. BLS can solve this problem in a different way, providing good universal approximation ability with a flat structure. The universal approximation ability of BLS has been proven by Chen et al. (2018). Our proposed method utilizes a convolution layer as a feature extractor to provide deep space features for BLS, and our proposed EBLS model can enhance the features in broad space before computing the final output. The comparison in the section above demonstrates that our method achieves better performance than state-of-the-art deep learning methods, which demonstrates the role of broad learning in feature enhancement. In addition, compared to other studies that have only used dozens of images, our model was trained and validated on a large dataset containing thousands of images and achieves better performance, which indicates that our method has better generalizability. However, in real clinical scenarios, the reasons for hypometabolism observed in FDG-PET may be more complicated. For instance, other types of dementia, such as dementia with Lewy bodies (DLB) or frontotemporal dementia (FTD), may also cause pathological changes similar to AD. Further studies that verify this method on more complex data may in future provide more reliable clinical aids for diagnosis of AD.

Our study also has limitations. First, although the machine learning method has achieved very good results in the validation with the ADNI data set, actual clinical prediction is much more complicated. For instance, many patients may have neurological diseases other than AD, which will affect the prediction results. We will continue our investigation and apply our model to a more general patient population in the future. Second, the algorithm can learn features that are difficult to see with the naked eye (which means that its predictions can differ from experts' interpretations), and t-SNE dimension reduction also shows the gradual progression of patients from MCI to AD, but the model cannot provide interpretable information for radiologists.

## 5. Conclusion

In conclusion, in our study we have developed a novel broad network-based model for prediction of AD diagnosis using 18F-FDG-PET of the brain. The proposed broad learning-based model was able to achieve high accuracy, sensitivity, and specificity on the validation set and outperformed professional radiologist readers in predicting AD based on FDG-PET. Moreover, the proposed model can be integrated into the clinical workflow as a powerful auxiliary diagnosis tool for reading PET imaging of patients with AD.

## Data availability statement

Publicly available datasets were analyzed in this study. This data can be found here: https://adni.loni.usc.edu.

## Ethics statement

Ethical approval was not provided for this study on human participants because the data used in this research was obtained from public available dataset. The patients/participants provided their written informed consent to participate in this study.

## Author contributions

JD: conceptualization, methodology, validation, visualization, writing—original draft, writing—review and editing, supervision, project administration, and funding acquisition. YL: data curation, methodology, software, validation, and writing—original draft. HW: validation and software. JW: supervision and project administration. LC: visualization and writing—review and editing. CC: conceptualization, resources, and supervision. All authors contributed to the article and approved the submitted version.

## Footnotes

^1^The code of EBLS model (https://github.com/YangLiuuuu/Extreme-Broad-Learning-System).
