# Supplementary material for: Broad learning for early diagnosis of Alzheimer's disease using FDG-PET of the brain
Source: Front Neurosci. 2023 Mar 13;17:1137567. doi: 10.3389/fnins.2023.1137567 (PMC10040750; doi:10.3389/fnins.2023.1137567)
Supplement: Supplementary file 1 [file Data_Sheet_1.docx]

Supplementary Material

Extreme Broad Learning System

# The algorithm of BLS

Despite strong learning ability of deep neural network, it is easy to overfit on small datasets and its training is very time-consuming. BLS is a lightweight network with broad structure. The inspiration of its design comes from random vector functional link neural network (RVFLLNN). It obtains global optimal solution by ridge regression algorithm during training without iterative back propagation process, so it’s training is very fast.

Assuming we have N training samples {X, Y} with X = [$X_{1}$,$X_{2}$, ...,$X_{N}$], where $X_{i}\in\mathbb{R}^{D}$, and $Y={[Y_{1}, Y_{2}, \ldots, Y_{N} ]}^{T}$, where $Y_{i}\in\mathbb{R}^{d}$, in which D and d is dimension of a single sample and output, respectively. The BLS firstly generates feature node $F_{i}$ with mapping function as follow.

$F_{i}=\phi_{i}(XW_{f_{i}}+\beta_{f_{i}})$, i=1, 2, …, n (1)

In the equation above, $W_{f_{i}}$, is weight matrix, and $\beta_{f_{i}}$ is bias vector, they are both randomly assigned, subscript $f_{i}$ indicates that it is the transformation weights of $i_{th}$ feature nodes, $\phi_{i}$ is activation function providing non-linear transformation. For convenience, we denote $F\equiv[ F_{1}, F_{2},\ldots, F_{n}]$ and assume $j_{th}$ enhancement node is $E_{j}$, we have

$E_{j}=\xi_{j}(FW_{e_{j}}+\beta_{e_{i}})$, i=1, 2, …, m (2)

Similarly, $W_{e_{j}}$ and $\beta_{e_{i}}$ are randomly generated matrix and bias, $\xi_{j}$ is activation function. We denote $E\equiv{[E}_{1}, E_{2},\ldots, E_{m}]$, then the input of hidden layer is $A=\left[ F | E \right]$. We have

$Y=\left[ F | E \right]W=AW$ (3)

where the weight of hidden layer W is calculated by pseudoinverse:

$W=\left[ F | E \right]^{+}Y=A^{+}Y$ (4)

Here, $A^{+}$is pseudoinverse of A, and

$A^{+}=\lim_{\lambda\to0} {(\lambda I+A^{T}A)}^{-1}Y$ (5)

# Extreme BLS

In standard BLS, additional feature nodes, enhancement nodes and input data can be added to the existing model without retraining the whole network when the accuracy is not as expected. We extended this incremental learning ability to a more generalized level by stacking groups of BLS blocks horizontally together. When a new BLS block is added, the residual between them would be minimized and the output of network would be closer to the ground truth.

Recall that in the standard BLS, the network takes the X as input and expects output Y. Assuming we have K standard BLS blocks, in order to differentiate different blocks, we reformulate Eq.(3) as follows.

$\rho_{j}=\left[ F^{j} | E^{j} \right]W^{j}=A^{j}W^{j}, j=1,2,\ldots,K$ (6)

where $\rho_{j}$ is the output of $j_{th}$ BLS block, $F^{j}$ and $E^{j}$ can have different number of nodes according to model complexity. Similar to Eq.(1) and Eq.(2), we use randomly initialized weight to get feature nodes and enhancement nodes of every block. For convenience, we also reformulate them by

$F_{i}^{j}=\Gamma\left( X,W_{f_{i}}^{j} \right), i=1,2,3, \ldots, n$ (7)

Then we have $F^{j}\equiv[F_{1}^{j},F_{2}^{j}, \ldots, F_{n}^{j}]$, and use it obtain enhancement nodes,

$E_{i}^{j}=\Omega\left( F^{j},W_{e_{i}}^{j} \right), i=1,2,3, \ldots, m$ (8)

where $W_{f_{i}}^{j}$ and $W_{e_{i}}^{j}$ are randomly initialized matrix and bias for generating feature nodes and enhancement nodes. So enhancement nodes of $j_{th}$ block is $E^{j}\equiv[E_{1}^{j},E_{2}^{j}, \ldots, E_{m}^{j}]$, and we use $A^{j}$ represent $\left[ F^{j} | E^{j} \right]$. When j = 1, the block is equivalent to standard BLS, which takes X as input, and Y as expected output. The hidden layer weight $W^{1}$ can derive by

$W^{1}= \lim_{\lambda\to0} {[\lambda I+A^{1}\left( A^{1} \right)^{T}]}^{-1}\left( A^{1} \right)^{T}Y$ (9)

When j > 1, the new BLS block will bring the output of the network closer to the ground truth. Firstly, the residual between the output of network and ground truth can derive by

$e_{j}=Y-\sum_{i=1}^{j} h(\rho_{i})$ (10)

where h(.) is any mapping function that bring more powerful non-linearity learning ability, for convenience, here we just choose identity function, so $h\left( \rho_{j} \right)=\rho_{j}$. We set the expected output of $j_{th}$ block as $e_{j}$, and the block take X as input. The block obtains feature nodes and enhancement nodes by Eq. (7) and Eq. (8). Specifically, we set $e_{1}=y$,, then we have

$W_{j}= \lim_{\lambda\to0} {[\lambda I+A^{j}\left( A^{j} \right)^{T}]}^{-1}\left( A^{j} \right)^{T}e$ (11)

where j=1,2,...,K. Keep adding new block until $e_{j}$< ϵ, if ϵ is very close to zero, we have

$Y-\sum_{j=1}^{K} h(\rho_{j})\approx0$ (12)

which is equivalent to

$Y\approx\sum_{j=1}^{K} h(\rho_{j})$ (13)

That is, the final output is the sum of all BLS blocks. Algorithm 1 is the procedure of our proposed EBLS.

## Supplementary Figures


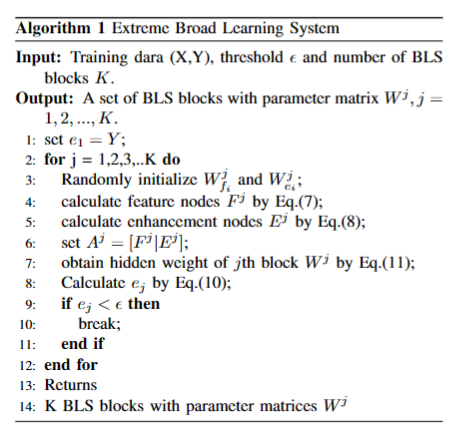


**Supplementary Figure 1.** The algorithm of EBLS
